# Supplementary figures and images for: Novel protein candidates for serodiagnosis of African animal trypanosomosis: Evaluation of the diagnostic potential of lysophospholipase and glycerol kinase from Trypanosoma brucei
Source: PLoS Negl Trop Dis. 2021 Dec 17;15(12):e0009985. doi: 10.1371/journal.pntd.0009985 (PMC8719729; doi:10.1371/journal.pntd.0009985)

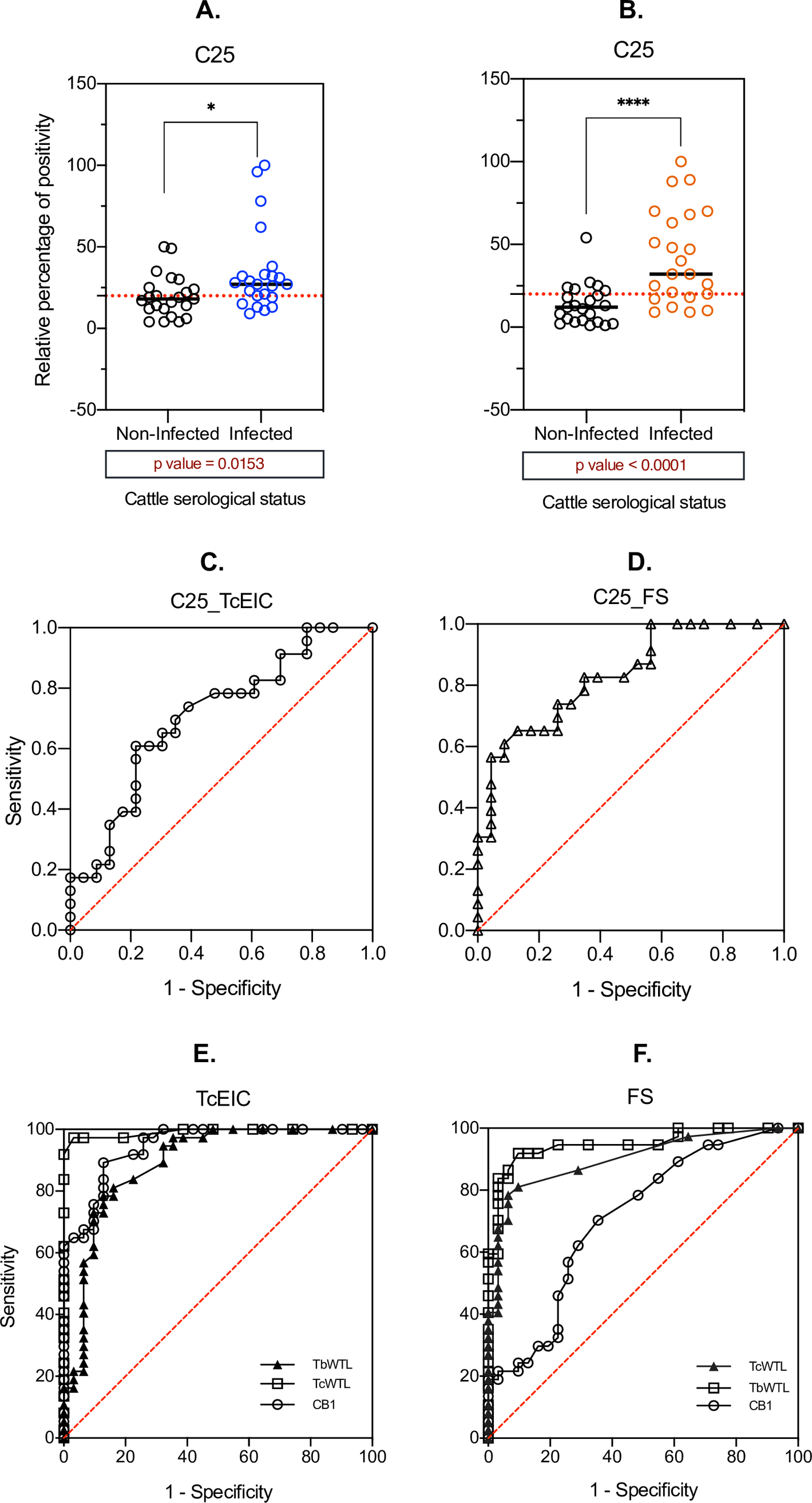

Supplement: S1 Fig — (A-B). C25 protein in single antigen Ab-ELISA test with experimentally T. congolense-infected bovine (TcEIC) sera (A) and field naturally T. congolense, T. vivax and T.b brucei infected bovine sera, field sera, FS (B). (C-F). ROC curve analysis: C25 (C-D), TcoCB1 and references antigens (TbWTL, TcWTL) testing with TcEIC (E) and FS sera (F). (TIF) [file pntd.0009985.s001.tif]

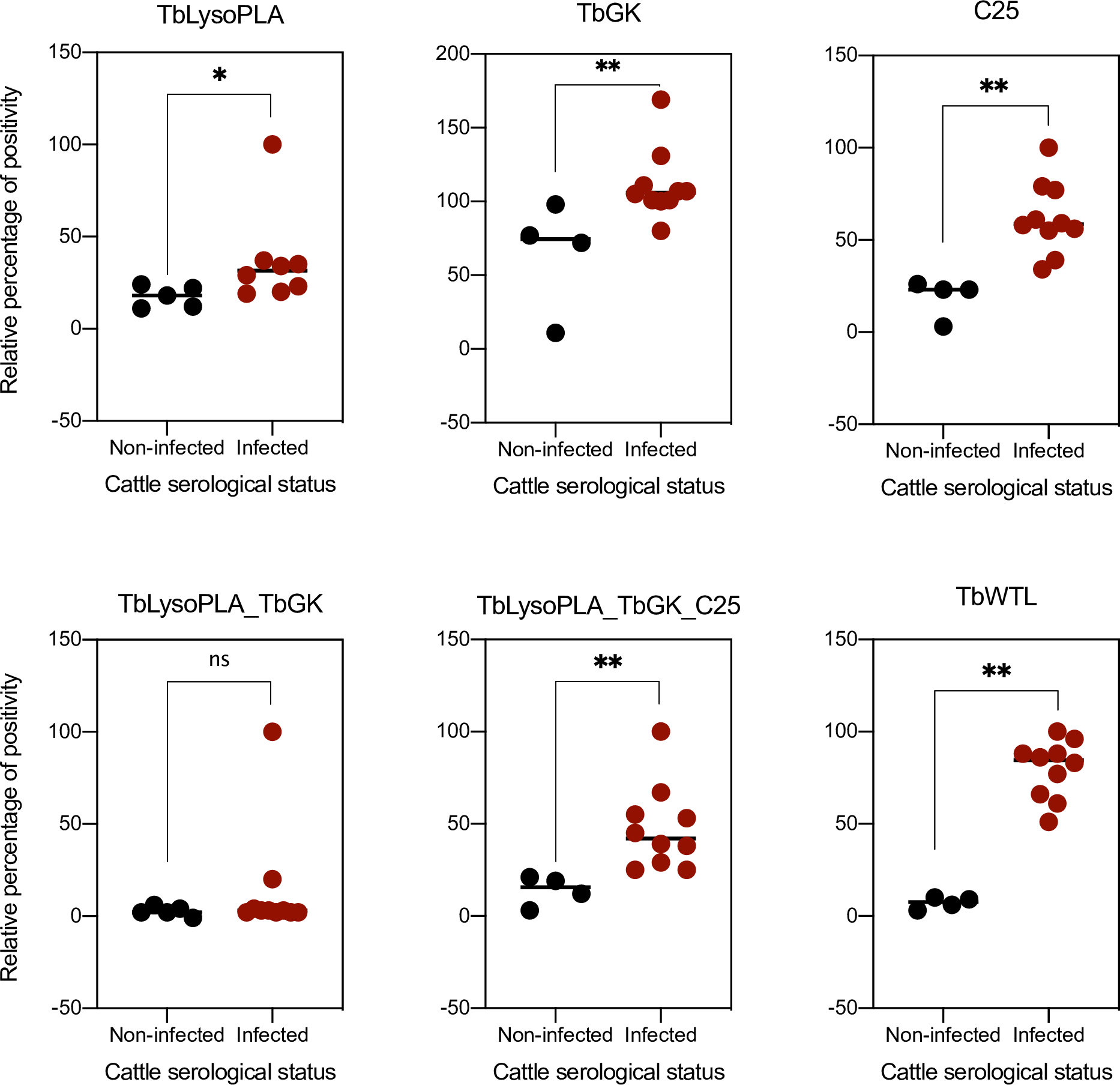

Supplement: S2 Fig — The figures represent the RPP medians values with their 95% CI values for infected and non-infected bovine sera. (TIF) [file pntd.0009985.s002.tif]

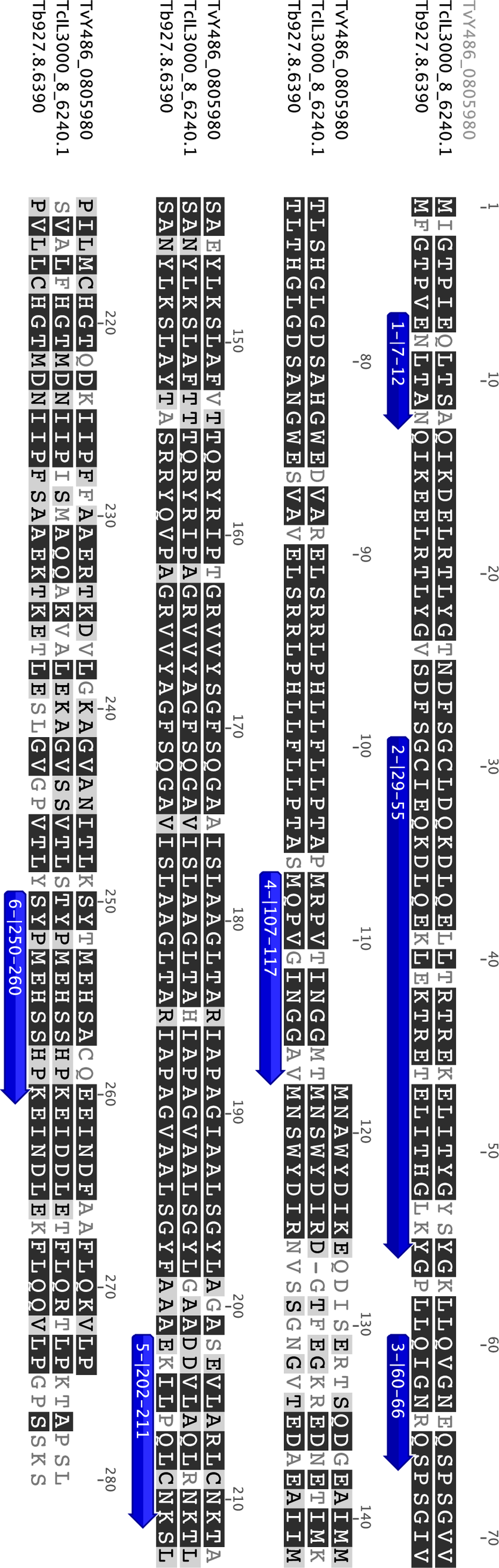

Supplement: S3 Fig — The IDs: Tb927.8.6390 (Trypanosoma brucei), TcIL3000_8_6240 (T. congolense), TvY486_0805980 (T. vivax). (TIF) [file pntd.0009985.s003.tif]

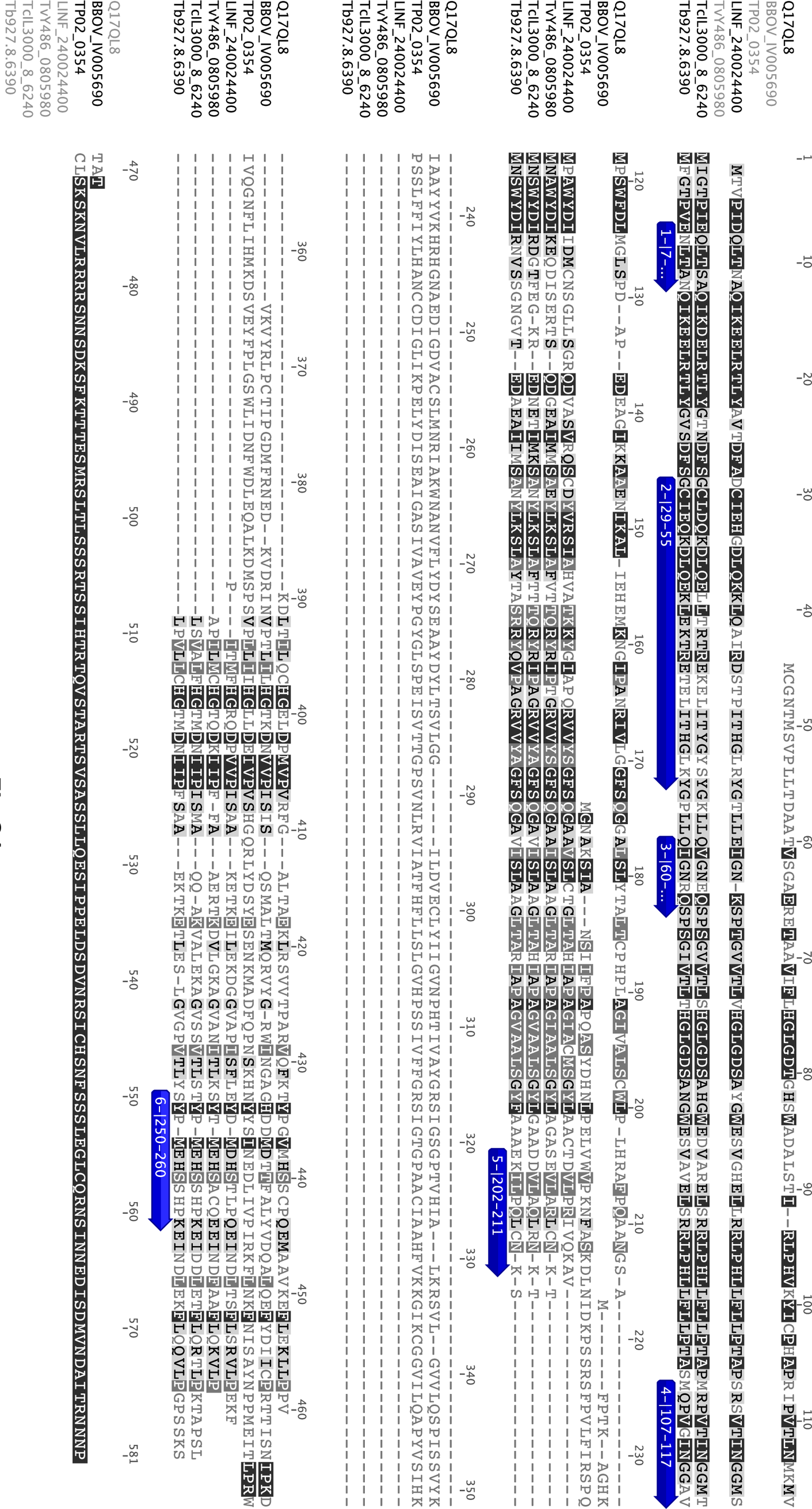

Supplement: S4 Fig — The IDs: Tb927.8.6390 (Trypanosoma brucei), TcIL3000_8_6240 (T. congolense), TvY486_0805980 (T. vivax), LINF_240024400 (Leishmania infantum), BBOV_IV005690 (Babesia bovis), TP02_0354 (Theileria parva), Q17QL8 (Bos taurus). The predicted epitopes in Table 2 for TbLysoPLA were annotated (by epitope number and position) on the protein sequence aligned with orthologs. (TIF) [file pntd.0009985.s004.tif]

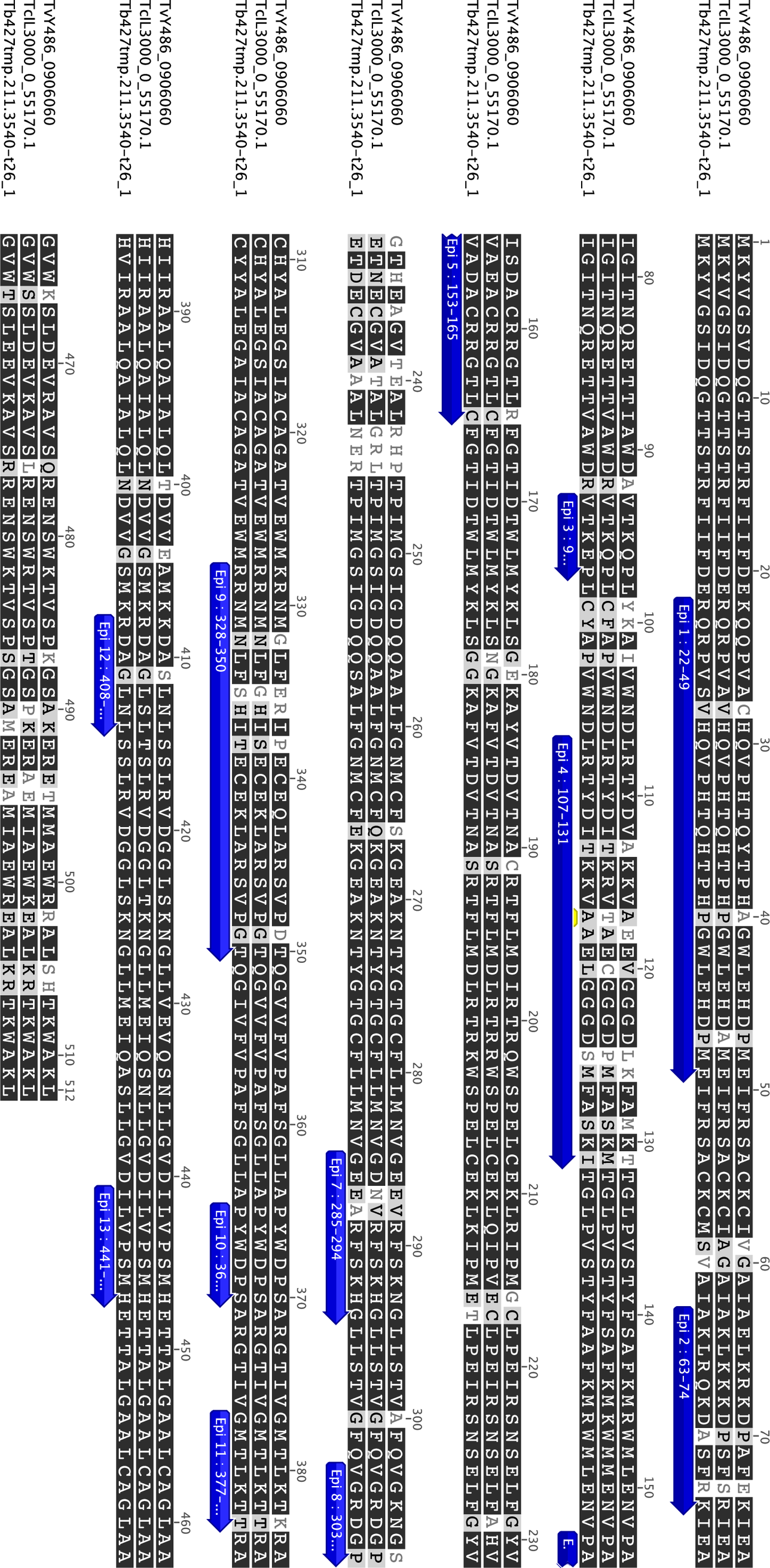

Supplement: S5 Fig — The IDs: Tb427tmp.211.3540-t26_1 (Trypanosoma brucei), TcIL3000_0_55170 (T. congolense), TvY486_0906060 (T. vivax). (TIF) [file pntd.0009985.s005.tif]

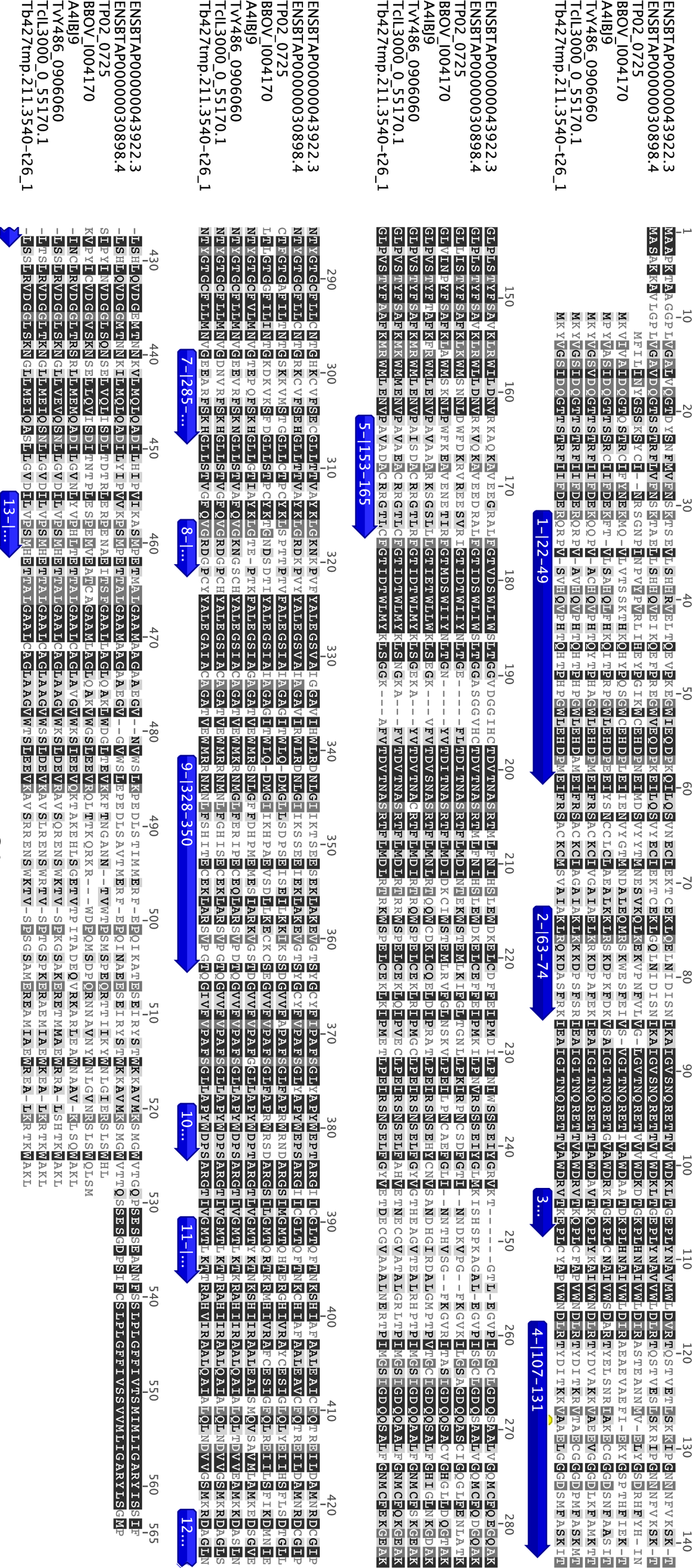

Supplement: S6 Fig — The IDs: Tb427tmp.211.3540-t26_1 (Trypanosoma brucei), TcIL3000_0_55170 (T. congolense), TvY486_0906060 (T. vivax), A4IBJ9 (Leishmania infantum), BBOV_I004170 (Babesia bovis), TP02_0725 (Theileria parva), ENSBTAP00000043922.3, ENSBTAP00000030898.4 (Bos taurus). The predicted epitopes in Table 2 for TbGK were annotated (by epitope number and position) on the protein sequence aligned with orthologs. (TIF) [file pntd.0009985.s006.tif]
