# Supplementary material for: Novel protein candidates for serodiagnosis of African animal trypanosomosis: Evaluation of the diagnostic potential of lysophospholipase and glycerol kinase from Trypanosoma brucei
Source: PLoS Negl Trop Dis. 2021 Dec 17;15(12):e0009985. doi: 10.1371/journal.pntd.0009985 (PMC8719729; doi:10.1371/journal.pntd.0009985)
Supplement: Attachment — Submitted filename: Tounkara_Responses to reviewers.docx [file pntd.0009985.s007.docx]

**Point-by-point reply**

We really appreciated the constructive comments our 3 reviewers and tried to improve the manuscript as much as we could. Please find below our answers (in colors) to each comment/question.

***Results***

Reviewer #2: Overall, the analysis presented matches the analysis plan, and results are clearly presented. I have only a few minor comments:

1. The rationale for choosing the two proteins as diagnostic candidates is currently outlined in the first two paragraphs of the results section. I would consider moving these to the end of the introduction as there are no real "results" to present regarding the identification of these two protein candidates (for example, TbLysoPLA is described as a secreted protein that induces production of specific antibodies, but these data are from a different study). This would make the protein sequence analysis the first paragraph of the Results section.

*Answer: We agree and moved this section to the introduction while maintaining a consistent message with the original manuscript.*

2. To give a more detailed overview of the two protein candidates, it may help to include protein sequence alignments of *T. brucei*, *T. congolense* and *T. vivax* for LysoPLA and GK (supplementary figures). These alignments can also be linked to the antigenic peptide sequences outlined in table 2, which would help the readers to see how similar these regions are between the three trypanosome species (in fact, it would greatly support the statement made on line 269, that these regions are conserved in all three species).

*Answer: We completely agree with this comment. We have made these alignments. As requested also by the reviewer #1, we have added some supplementary figures (see the answer to reviewer #1 comment 3).*

3. This reviewer is not very familiar with the BepiPred-2.0 tool. In table 2, what is the rationale for the different threshold parameters (0.52 and 0.45 for TbLysoPLA and TbGK, respectively), and should these not be the same?

*Answer: The epitope prediction is based on protein proprieties (hydrophobicity, flexibility, accessibility, polarity etc..). The default threshold recommended by the prediction algorithm is 0.5. The threshold choice for one protein is independent to another one as long as we do not compare the immunogenicity of these two proteins. we chose a lower threshold for TbGk because we observed either very short (less than 5aa) or very long (more than 30aa) epitopes for TbGk at the default threshold. For TbLysoPL at the default threshold or at the 0.52 threshold, the results were the same. We selected a higher threshold to show that all the predicted epitopes had a high immunogenic profile.*

4. For clarity, would the authors be able to include Gene IDs for the TbLysoPLA and TbGK genes in the methods (currently TbLysoPLA only) and/or results sections (e.g. Table 2). It would also be beneficial to the reader to include gene IDs for the orthologs in T. congolense and T. vivax.

*Answer: We have included all the Ids (see Fig. S3, S4, S5 and S6).*

5. Do the authors have access to any other parasitaemia data for the samples used in this study, especially for the experimental samples? This would give an idea of the accuracy of the serodiagnostics analysis in comparison to conventional diagnostics such as microscopy.

*Answer: It would indeed have added value to our analysis if we had compared the data, but unfortunately, we do not have access to the parasitemia data.*

6. Figure 1B - Whilst RNAi of TbGK clearly leads to a reduction in protein (~55 kDa), the PFR control is also significantly reduced. Can the authors explain this?

*Answer: The reduction in PFR control in the RNAi extract could be explained by the fact that the amount of total protein loaded in the gel is lower compared to the one loaded for WT. We did not repeat this western blot because the objective was just to point-out the specificity of our antibody.*

7. Line 420 - I believe PLOS NTDs does not accept references to "Data not shown" - therefore the data regarding shared epitopes of TbLysoPLA between T. brucei and T. congolense will need to be included, if available (perhaps in supplementary materials?).

*Answer: We have taken this recommendation into account and have added sequence alignments showing the predicted epitopes for each protein as supplementary figures (answer to comment 2).*

Reviewer #3: Please see summary below

I. **Conclusions**

Reviewer #2:

1. One aspect that is not discussed is whether these serodiagnostic candidates are also effective in diagnosing all three subgroups of T. congolense (Savannah, Forest and Kilifi), could the authors comment on this (I assume, perhaps wrongly, that T. congolense IL3000 was used in the experimental samples)?

*Answer: The strain used in the experimental samples was the T. congolense IL1180. In regard to the effectiveness of the present protein candidates to diagnose the other subgroups of T. congolense, we do not have more precise data because we have not specifically tested positive sera to these strains. However, the objective being to make a pan-trypanosome diagnosis and considering the reactivity of these proteins with TcIL1180 positive sera (which is very close to the other strains from a genomic point of view), we can suppose that a serodiagnostic test based on these candidate proteins could also detect these other T. congolense strains.*

2. Can the authors comment whether there is cross-reactivity with Trypanosoma theileri infections? This species is known to infect a range of bovine hosts, but is not always clinically relevant (PMID: 28903536).

*Answer: It is not known and “cross-reactivity has been poorly investigated. We have no evidence that there is cross-reactivity with T. theileri. But if we consider the sequence identities of our candidate proteins and theirs orthologs in T. theileri, it seems quite likely that there is a possible cross-reactivity. However, this needs to be confirmed experimentally by directly testing T. theileri positive bovine sera on the candidate proteins.*

*TbLysoPLA vs TtLysoPLA (*TM35_000152590) *[identity 65%]*

```
Query  1    MFGTPVENLTANQIKEELRTLYGVSDFSGCIEQKDLQEKLEKTRETELITHGLKYGPLLQ  60
            M GTP+ENLT  QI++ELR LYGV DFS C+E KDLQ+KL K R+TE ITHGL+YGPLLQ
Sbjct  1    MIGTPIENLTLAQIRDELRNLYGVKDFSDCLELKDLQDKLTKVRDTEPITHGLRYGPLLQ  60

Query  61   IGNRQSPSGIVTLTHGLGDSANGWESVAVELSRRLPHLLFLLPTASMQPVGINGGAVMNS  120
            IGN+QSP+G+VTL+HGLGDSA+GWE VA E+SRRLPHLLFLLPTA ++PV INGG  MN+
Sbjct  61   IGNKQSPTGVVTLSHGLGDSAHGWEDVASEISRRLPHLLFLLPTAPVRPVTINGGMSMNA  120

Query  121  WYDIRN-VSSGNGVTEDAEAIIMSANYLKSLAYTASRRYQVPAGRVVYAGFSQGAVISLA  179
            WYDI N + + +   +++EA++ SA+Y+ SLAY A++RY +P  RVVYAGFSQGA ISLA
Sbjct  121  WYDITNIIGTASPPPQESEAVLASADYICSLAYNATQRYHIPKNRVVYAGFSQGAAISLA  180

Query  180  AGLTARIAPAGVAALSGYFAAAEKILPQLCNKSLPVLLCHGTMDNIIPFSAAEKTKETLE  239
            AGLT RI  AG   LSGY A   +IL +L NK++P+ +CHGT D I+PF AAE+TKE L+
Sbjct  181  AGLTGRIPAAGAVVLSGYLAGGNEILSRLRNKNVPIFMCHGTQDTIVPFDAAERTKEALK  240

Query  240  SLGVGPVTLYSYPMEHSSHPKEINDLEKFLQQVLP  274
              GV  V L SYPMEHSSHPKEI D+ KFL+QVLP
Sbjct  241  KAGVSSVKLNSYPMEHSSHPKEIEDVVKFLKQVLP  275
```

*TbGK vs TtGK (TM35_000043700) [70%]*

```
Query  1    MKYVGSIDQGTTSTRFIIFDERQRPVSVHQVPHTQHTPHPGWLEHDPMEIFRSACKCMSV  60
            MKY+G++DQGTTSTRFIIFDERQR V+ +QVP TQ+TP PGWLEHDPMEIF+ +CKC+
Sbjct  1    MKYIGALDQGTTSTRFIIFDERQRLVARYQVPITQYTPQPGWLEHDPMEIFKVSCKCIVA  60

Query  61   AIAKLRQKDASFRKIEAIGITNQRETTVAWDRVTKEPLCYAPVWNDLRTYDITKKVTAEL  120
            A+A+LR KD SF K+EAIGI NQRETTVAWDR TK+PL  A VWNDLRTY +TK++  +L
Sbjct  61   AVAELRSKDPSFDKLEAIGIANQRETTVAWDRETKKPLYNAIVWNDLRTYGVTKEIAEKL  120

Query  121  GGGDSMFASKITGLPVSTYFAAFKMRWMLENVPAVADACRRGTLCFGTIDTWLMYKLSGG  180
            GGGD  FA +  GL  STYF+AFKMRWML+NVP VA+A +RG+LCFGT+D+WLM+ LSGG
Sbjct  121  GGGDIRFAIQKNGLLASTYFSAFKMRWMLDNVPDVAEANKRGSLCFGTVDSWLMWMLSGG  180

Query  181  KAFVTDVTNASRTFLMDLRTRKWSPELCEKLKIPMETLPEIRSNSELFGYVETDECGVAA  240
              F+TDVTNASRTFLMD+RTR WS ELCEKL IPM++LPEIRSNSE F ++ +DE G+A+
Sbjct  181  SCFLTDVTNASRTFLMDIRTRSWSSELCEKLGIPMKSLPEIRSNSEFFTHILSDEAGLAS  240

Query  241  ALNERTPIMGSIGDQQSALFGNMCFEKGEAKNTYGTGCFLLMNVGEEARFSKHGLLSTVG  300
            AL  +TPIMG IGDQQ ALFGNMCF +GEAKNTYGTGCFLLM VGEE RFS+HGLLSTV
Sbjct  241  ALRHKTPIMGCIGDQQGALFGNMCFNQGEAKNTYGTGCFLLMVVGEEVRFSQHGLLSTVA  300

Query  301  FQVGRDGPCYYALEGAIACAGATVEWMRRNMNLFSHITECEKLARSVPGTQGIVFVPAFS  360
            FQ+G++G C YALEG+IA AGA +EWM+ N+ LF +IT+CEK+ARS+P TQG+VFVPAFS
Sbjct  301  FQLGKEGSCTYALEGSIAGAGAAIEWMKDNLKLFKNITDCEKIARSIPDTQGVVFVPAFS  360

Query  361  GLLAPYWDPSARGTIVGMTLKTTRAHVIRAALQAIALQLNDVVGSMKRDAGLNLSSLRVD  420
            GLLAPYWDP+ARGTI+GMTLKTT AH++RAAL+AIALQ+ DVV +MK+DA LN+  LRVD
Sbjct  361  GLLAPYWDPTARGTILGMTLKTTNAHIVRAALKAIALQVVDVVEAMKQDAELNVEMLRVD  420

Query  421  GGLSKNGLLMEIQASLLGVDILVPSMHETTALGAALCAGLAAGVWTSLEEVKAVSRRENS  480
            GGLS N LLME+QA LLGVD+ +P+M ETTALGAALCAGLAAGVW SL+EVKAV+  E+
Sbjct  421  GGLSGNRLLMEMQAGLLGVDLRLPAMAETTALGAALCAGLAAGVWKSLDEVKAVAAEEHK  480

Query  481  WKTVSPSGSAMEREAMIAEWREALKRTKWAKL  512
            W+ + P  S+  R  +  +W+ AL++ KWAKL
Sbjct  481  WEVIKPKVSSEFRREIKEQWKRALQKAKWAKL  512
```

3. From this study, it appears that the use of at least two proteins is a significant improvement on diagnostics, compared to using an individual protein. Is there an optimum number of proteins to include in a panel for serodiagnosis (i.e. if a third protein was included, would this improve the diagnostic capability even more)?

*Answer: This is a very interesting question. We have seen that combination of more than three proteins decrease the diagnostic performance of the proteins cocktail. This observation was made in a study performed on 3 other proteins in the context of the PhD project of Magamba Tounkara. In this study (preliminary data), the cocktail of 4 proteins showed lower specificity compared to a 2 or 3 protein cocktail. However, it should be noted that the proteins tested in this study were either of low putative immunogenicity or low purity. This question will clearly be addressed by the follow-up of this study and the answer will be useful in the design of the protein mixture for a commercialized test.*

4. The authors provide a good discussion of the importance of novel serodiagnostics candidates, but I was wondering whether they can discuss any other protein candidates that may be suitable in addition to the two outlined in this study (for example, by taking into account previous secretome studies of T.b. brucei)?

*Answer: It is true that the secretome could be a source of candidates for serodiagnosis. We didn’t want to discuss this point in this manuscript because it is a vast topic and we really wanted to focus the attention on our two candidates and on the use of a combination of these molecules.*

5. Public health relevance is nicely addressed in the discussion section - especially the point that a comprehensive test encompassing the three main pathogenic trypanosome species is highly desirable.
II. **Editorial and Data Presentation Modifications?**

Reviewer #2: There are several minor grammatical and/or spelling errors to address, as well as some missing references in the introduction:

Line 60 – A very recent study found that vaccination against T. vivax is possible using an invariant surface protein antigen (PMID: 34040257). This should be included in the introduction.

*Answer: We have mentioned and included this study in the introduction.*

Line 66 – Parasite-derived RNA can be detected during in vivo infections, as well as DNA and proteins (PMID: 30779758).

*Answer: We fixed this.*

Line 68 – ….comparatively easy to perform but lacks sensitivity (missing the ‘s’ after lack).

*Answer: We fixed this.*

Line 84 – Protein mixtures.. (don’t need the ‘s’ at the end of proteins).

*Answer: We fixed this.*

Line 99 -  Where applicable, it would be useful for the reader to know which strains were used for each species (e.g. T. congolense I assume is IL3000 and T. vivax is Y486?). Same goes for Table 1, for trypanosome strains.

*Answer: We fixed this.*

Line 107 – There is a space after TcoCBP1 before the closing bracket that is not needed (also on line 337).

*Answer: We fixed this.*

Line 146 – “As regards the TbGK protein, it was purified…”, consider revising (e.g. “With regards to the TbGK protein”)

*Answer: We fixed this.*

Line 406 - "two novel proteins" - consider revising because the proteins themselves aren't novel, but as candidates for serodiagnosis they are novel.

*Answer: We fixed this.*

Line 469 - "are way more sensitive" -> "exhibit increased sensitivity"

*Answer: We fixed this.*

**V. Summary and General Comments**

Reviewer #1: Review of Tounkara et al.,  “Novel protein candidates for serodiagnosis of African animal trypanosomosis:  Evaluation of the diagnostic potential of lysophospholipase and glycerol kinase from Trypanosoma brucei”

In this manuscript, Tounkara and colleagues describe the assessment of two proteins from Trypanosoma brucei as candidate components of a serology-based diagnostic approach for use in animal disease surveillance and treatment campaigns.  The two proteins, a lysophospholipase A and glycerol kinase, are immunogenic and generally abundant, suggesting that they would be reasonable candidates for such a tool.  While the individual proteins alone performed poorly, while the two combined with a third previously described antigen were more promising.  While none of the combinations reached the level of sensitivity of total antigen samples, the work does provide evidence that the right cocktail of recombinant antigens could be identified to yield a standardized and sensitive assay for use in animals.

A. Major Concerns:

1. Line 72 – The sentence starting on line 71, “However, there is as yet no…, that would allow detecting active infections” is a little misleading.  Serology-based tests often cannot distinguish between current and previous (particularly, previously treated) infections.  This is antigen/antibody dependent but may limit he utility of a serological in treatment campaigns involving domesticated animals that may have multiple infections in a lifetime.  Some comment should be included about this potential limitation.

*Answer:* Indeed, antibody-based tests are especially useful for epidemiological purposes. Because of the persistence of antibodies in the blood for several months after spontaneous recovery or after treatment, they do not indicate only active infection but merely the contact, present or past, with the parasite. Therefore, serology-based antibody detection is more suitable for herd level diagnostic. Despite this limitation it’s still the easiest test adaptable to rapid diagnostic (the easiest test to use in field condition). The recommendation will be taken into account.

2. Line 237 – The function of GK in trypanosomes is not entirely resolved.  It is essential in BSF parasites grown in glycerol-rich media, but that may not be its only function given its abundance. To suggest it is a glycolytic enzyme is not exactly accurate – it is involved in anaerobic glucose metabolism but is not a classical component of glycolysis.

*Answer:*  We agree with this comment. This part has been re-phrased to facilitate the understanding of the document.

(*to be included*) It has been shown that GK of *T.b. brucei* is expressed in large excess in BSF. By maintaining a high level of glycerol metabolic flux, the GK become essential for BSF (Pineda *et al*., 2018). The GK is glycosomal and represent with known glycolytic enzymes up to 4% of the trypanosome proteome.

3. Lines 264-269.  It is unclear if the predicted epitopes are unique to the trypanosome proteins – some comment on this would enhance the value of this analysis.

*Answer:* In fact, we didn’t include the supplementary data on protein sequences analysis in the document. It appears to be important to include those data. In order to meet the request of reviewer 2, we have added the results of the protein sequence alignments (*Fig. S3, S4, S5 and S6*). For each alignment, the predicted epitopes have been annotated at the position they have been predicted. In regard to this analysis, the predicted epitopes for TbLysoPLA are conserved and unique to trypanosome proteins when compared to its orthologs presented in our analysis. In contrast, the predicted epitopes for TbGK are not all unique to trypanosome proteins. The predicted epitope 10 is conserved in *Leishmania* proteins (*Fig. S5, S6*).

4. What do the tops of the blots in Fig 1B look like.  They seem to be cropped differently than the other blots and gels.

*Answer:* The tops of those blots were cut and incubated with the anti-PFR antibody before the revelation. The PFR protein sizing up to 74 KDa has been used as loading control.

5. Fig. 4 – Some comment should be added to the text to address the difference in performance of the three-protein cocktail when the experimentally infected vs. field sera are analyzed

*Answer:* This is a very important recommendation. The main difference in performance of the three-protein cocktail between experimentally infected and field sera is that field infected cattle sera can present antibodies against the three different trypanosomes species. Most of the epitopes of the mixture are targeted by these antibodies, the performance of the protein cocktail is better. In contrast, in experimentally infected cattle sera, antibodies targeting only *T. congolense* epitopes conserved in trypanosomes are present. This explains why the cocktail has a better performance with field sera.

B. Minor Comments:

1. Abstract, line 35 – insert “other” before trypanosomes :

*Answer: We fixed this.*

2. Line 59-60 – change to  “to regularly change their glycoprotein coat, conventional vaccines have not yet been developed…”

*Answer: We fixed this.*

3. Line 285 – (needs two commas) “…hexa-histidine tag, respectively, were…”

*Answer: We fixed this.*

4. Line 502 – should be “Fig. 5” not “Fig. 4”

*Answer: We fixed this.*

6. Figs. 3 and 4 – “pourcentage” should be changed to percentage (or %) in the Y axis labels.

*Answer: We fixed this.*

Reviewer #2: See above for my overall comments. This is an interesting study providing new candidates for the development of serodiagnostics to identify active AAT infections, which are sorely needed for animal trypanosomiasis. I feel a few minor details are required, but certainly no extra experiments.

Reviewer #3: This study by Tounkara et al. evaluates the potential of two recombinantly expressed T. brucei proteins, individually and in combination with the C25 antigen, for the serodiagnosis of African animal trypanosomosis (AAT). The authors show that, overall, in combination these antigens show improved performance in ELISA assays.

The data presented here are of a very high technical standard and will be useful for researchers in the area of AAT diagnostics. Some issues with data presentation and interpretation need to be addressed before publication, however.

1) When comparing conservation between orthologs, it is usually unclear if the authors are talking about percent identity or similarity (e.g. abstract, lines 257ff, lines 417ff). Please clarify this throughout the manuscript.

*Answer: Here we are talking about the percentage of identity. The misunderstanding has been clarified in the document.*

2) The authors may wish to qualify their comments on the potential of developing a vaccine against AAT (PMID 34040257)

*Answer: Different comments have been included about this new vaccine in the manuscript.*

3) Line 82: please provide references for C-25

*Answer: We fixed this.*

4) Line 83: “...initially selected from the literature for their immunogenicity” - please provide references

*Answer: The choice of the protein candidates has been moved to the introduction part with all the references.*

5) Line 110: please provide the systematic TriTrypDB ID for BiP/C-25

*Answer: We fixed this.*

6) Line 123: please provide the systematic TriTrypDB ID for TbGK

*Answer: We have included the TriTrypDB ID: Tb927.9.12630.*

7) Lines 129ff: please clarify which procedures apply to TbLysoPLA vs. TbGK. As written, the text suggests in both cases lysates were loaded onto a glutathione sepharose column.

*Answer: TbLysoPLA was purified using glutathione sepharose column and TbGK using affinity chromatography on the HisPur™ Ni-NTA nickel resin (Thermo Fisher Scientific, Waltham, MA, USA) according to the manufacturer's instructions.*

8) Line 181ff: Origin of the sera. According to the Methods, for the field samples it was known which trypanosome species an animal had been infected with. However, throughout the study this information is not being considered, although this might be very useful in assessing cross-reactivity. Please reconsider this decision, or discuss briefly why this information was not being taken into account. Perhaps sample sizes would have been too small to give statistically robust data?

*Answer: The sample status was considered in all analysis. The aim was to show that we can use recombinant protein for one species (here T.b. brucei) to detect other trypanosomes species (eg T. congolense and T. vivax). Species-specific data analysis from field sera would have reduced the sample size. Moreover, animals' status was determined mainly by PCR whereas i) PCR does not allow to confirm that an animal is not co-infected by several species of trypanosomes, ii) PCR does not give information on the presence of persisting antibodies reacting against a previous infection. In addition, the cross-reaction with trypanosome-infected bovine sera from the field, was expected.* *For these reasons, we did not conduct the analysis based on sample status for a single species.*

9) Line 229: “Phospholipases are among the enzymes...” - please provide references.

*Answer: We fixed this.*

10) Line 231: reference 15 seems wrong here

*Answer: We fixed this.*

11) Table 1: the first column should read (row 1) TbLysoPLA, (row 2) TcLysoPLA, (row 3) TvLysoPLA, (row 4) TbGK, (row 5) TcGK, row 6 (TvGK). Also, the much lower conservation (0.2%) between TvLysoPLA and the Theileria ortholog compared to TcLysoPLA seems odd. Is this a typo?

*Answer: it's an error in fact. The conversation is about (pairwise identity) 20% but there was only 0.4% of identical sites because the T. vivax ortholog is short.*

12) Line 266, immunogenicity scores: for those readers not familiar with these predictions, please provide context for these scores. Also, what do these findings imply or suggest with respect to the goal of the project?

*Answer: The goal is just to have an idea about the immunogenicity of the amino acid sequence from their intrinsic properties. We answer about this comment in the reviewers 1# and to #2 requests. Basically, the bio-informatic tool used predicts a peptide immunogenicity based on proprieties such as hydrophobicity, accessibility, flexibility in the global protein sequence etc. The details of the score calculation are not provided but there are several documents on the methods used on the IEDB site. In order to not distort our initial message, we have focused on the evaluation of the immunogenicity of the candidate proteins at the experimental level as shown in the different antibody detection tests.*

13) Line 264ff / Table 2: it would be very useful to provide detailed information on the conservation of the predicted epitopes between the trypanosomatids and between T. brucei and Bos bovis

*Answer: We added sequences alignments on supplemental informations.*

14) Lines 270/275: “Specific anti-TbLysoPLA...” - assessing specificity is the aim of this experiment

*Answer: The aim was to shown that we can use T.b brucei protein to detect T. congolense specific antibodies by cross-reaction.*

15) Lines 287 / 290: should that read “5 L” instead of “5 mL”?

*Answer: 5 mL*

16) Line 288 / Fig. 2A: if I understand correctly, TbLysoPLA was released from the column by cleavage with thrombin. Why is that protein (presumably ~36 kDa) not visible in the eluate?

*Answer: The amount of thrombin added is very low compared to the eluted TbLysoPLA and likely undetectable (6 units were added from an initial concentration of more than 50 units/mg of protein, i.e. about 0.1mg). It is quite possible that the thrombin signal was masked by TbLysoPLA which could reach up to 2 mg/mL in an eluate. Moreover, the presence of thrombin would not have been a problem for the different ELISA tests because it is a bovine protein.*

17) Line 295 (and elsewhere): according to the Methods section,

18) Line 308 (similar statements are being made elsewhere, e.g. lines 312, 341, 444): “...demonstrating that TbLysoPLA discriminates between infected and non-infected cattle”. As written, this is misleading. The statement is true for discriminating between groups of infected vs. non-infected cattle, but for individual animals (as indicated by poor specificity and sensitivity) it is not. Please revise these statements to avoid ambiguity.

*Answer: Yes, it can be misleading when we take the individual status of sera. But the message here was about the groups of positive and negative sera. We have corrected this misleading.*

19) Line 336: please give systematic TriTrypDB ID for TcoCB1. TcoCB1

*Answer: TcoCB1 has been included in a rapid diagnostic test (VerYDiag) by CEVA santé animale. However, in the two publications (Mendoza et al., 2008; Boulangé et al., 2018) regarding this protein, TritrypDB ID was not mentioned. Therefore, we cannot provide it unfortunately*

20) Line 377: “...TbLysoPLA and TbGK showed very high Sp values at 96.8% and 93.5%, respectively”. These values seem inconsistent with the data presented in Fig. 3C. If this was true, it should be possible to find a cut-off above which more than 90% of samples belong to the ‘infected’ category. The figure does not seem to support this.

*Answer: All the cut-off values were generated by the same method to test the performance of the different antigens for experimental and field sera (“*The cut-off value for each serum panel was determined using the method described by Johnson (2004) based on the likelihood ratio closer to the maximum vertical distance method [24]”*). We didn’t set a cut-off by hand for each test. It would have distorted the results if cut-offs had been established differently. Thus, the specificity values (as a reminder, represent TN/TN+FP) obtained from the computation at the cut-off presented, are quite logical.*

21) Line 387: “...AUC value increased for the proteins (sic) combinations as shown in Fig 4B-4D”. This is not the case for the +C25 samples in 4B (Table 4), which have a lower AUC value compared to the -C25 sample.

*Answer: Yes, that’s true. But it’s still increased compared to the individual protein AUC value.*

22) Line 412: “...to levels comparable to reference tests”. This is an overstatement. As acknowledged elsewhere, whole cells lysates consistently performed better.

*Answer: We fixed this.*

23) Line 446: “...showed AUC values of 0.78 to 0.86 higher than...” should read “...showed AUC values of 0.78 to 0.86, higher than...”, otherwise this is misleading.

*Answer: We fixed this.*

24) Line 453ff: this is a good paragraph on limitations of the study. Can you please comment on potential of cross-reactivity with Leishmania, considering the degree of protein conservation? Presumably this is not a big concern in cattle?

*Answer: We think that the cross-reaction is possible as well as for Trypanosoma theileri (asked by reviewer #2). More than 50% of conservation is quite consistent. If the predicted epitopes (on the tested protein sequences) are also conserved with the orthologs in leishmania, there is a high probability that the antibodies will cross-react. But, as you mentioned, leishmania infection in cattle doesn’t’ have a high veterinary importance as Piroplasma or Trypanosoma.*

25) Line 502. Fig. 5 legend mislabelled as Fig. 4.

*Answer: We fixed this.*

26) Fig S2: panels B-F are not referred to anywhere in the manuscript. Also, why are the data presented differently from other figures? Please show individual data points, as in Figs. 3 and 4.

*Answer: The Fig S2 was presented in this way to show that even if the number of sera is low, there is a pattern that shows the difference between infected and non-infected cattle groups.*
