# Supplementary material for: Novel protein candidates for serodiagnosis of African animal trypanosomosis: Evaluation of the diagnostic potential of lysophospholipase and glycerol kinase from Trypanosoma brucei
Source: PLoS Negl Trop Dis. 2021 Dec 17;15(12):e0009985. doi: 10.1371/journal.pntd.0009985 (PMC8719729; doi:10.1371/journal.pntd.0009985)
Supplement: Attachment — Submitted filename: Responses-to-reviewers_round2.docx [file pntd.0009985.s008.docx]

**Revision round 2 : Point-by-point reply**

Please find below our answers to reviewers 1 and 3.

**Reviewer #1:** Review of Resubmission of  Tounkara et al.,  “Novel protein candidates for serodiagnosis of African animal trypanosomosis:  Evaluation of the diagnostic potential of lysophospholipase and glycerol kinase from Trypanosoma brucei”

Overall, the authors have addressed the concerns I had with the manuscript.  Just a few very minor clarifications/edits to recommend below:

1.	The text found on lines 450-458 of the discussion is a bit confusing.  “In silico, the two proteins showed …and low homology with bovine (<35%) and Piroplasma (<15%)” seems to conflict with line 458, “…TbGK shares a high identity with both cattle (58%) and Piroplamsa (45-55%)”.  Please clarify this.

*Answer: We clarified this by adding this sentence (in blue in track version) : “In silico*, both TbLysoPLA and TbGK proteins showed strong identities with their orthologs in trypanosomes (over 60% for TbLysoPLA and over 82% for TbGK). They displayed low homology with bovine (<35%) and *Piroplasma* (<15%) orthologous proteins”. (line 487)

2.	Line 112 – please change, “By maintaining...” to “When maintaining…”

*Answer: We have made this modification.*

**Reviewer #3:** My comments have been addressed to my satisfaction, with two exceptions. I find the stated reason for not being able to provide the systematic ID for TcoCB1 (point 19) very odd, considering that the first author of one of the original studies is second author on this manuscript. And I still don't understand how the data shown in Fig 3C are consistent with the stated specificities (point 20). But considering that all the other comments have been addressed, I'll leave it to the Editor to decide is another round of revisions is necessary.

***Answer to the comment 19:***

*The reason why we could not provide the systematic ID of TcoCB1 can indeed appear odd. The original paper on TcoCB1 was published under a CEVA funded project. CEVA is a private company and they developed a commercial test out of this paper’s results. We wondered if we had the right to mention this ID in our study, as they did not do it themselves. At any rate, this protein was used only as a control.*

***Answer to the comment 20:***

*We agree that it is a bit difficult to understand by simply looking at the graph. But as we explained before (revision round 1), the cut-off has been established by a modeled method to allow comparisons.*

*Since the specificity (Sp) and sensitivity (Se) values are calculated using this cut-off, the Sp or the Se can be high even if the overall performance is weak. In Figure 3C, the high cut-off values (28 for TbLysoPLA and 50 for TbGK) classify most samples from non-infected animals as negative, and therefore as true negatives. Knowing that the Sp value represents TN/(TN+FP) [TN=true negative, FP=false positive], we obtain a very high Sp value. The Se being weak, this high Sp value does not bring any performance to the test. This is why we have provided the AUC values to better appreciate the global performance of the different tests.*
